# Invited commentary for surgery open: “Defining the feasibility of same day adrenalectomy - A prospective matched cohort study”

**DOI:** 10.1016/j.sopen.2023.09.010

**Published:** 2023-09-12

**Authors:** Anastasia K. Bogdanovski, Benjamin C. James

**Affiliations:** Harvard Medical School, Boston, MA, USA; Department of Surgery, Beth Israel Deaconess Medical Center, Boston, MA, USA

Laparoscopic adrenalectomy has become the standard surgical approach since it was first reported over three decades ago [1,2]. It has been shown to have fewer complications, lower mortality, and shorter length of stay than the open approach, and has thus been adopted as the standard of care [1,3,4]. However, most data on the safety and efficacy of laparoscopic adrenalectomy has been collected retrospectively. In “Defining the feasibility of same day adrenalectomy - a prospective matched cohort study,” the authors seek to address these gaps in knowledge by assessing outcomes prospectively while also establishing feasibility for same-day discharge pathways in patients undergoing laparoscopic adrenalectomy.

The authors hypothesize that outcomes following same-day discharge in patients undergoing laparoscopic adrenalectomy will be non-inferior to outcomes following planned postoperative admission. The study carefully selected ten patients who were slated to undergo adrenalectomy for primary hyperaldosteronism, Cushing syndrome, or non-functional adrenal nodule. These patients were then matched to a control group who those admitted after surgery and were followed for 30 days postoperatively. The primary outcome was “uncomplicated same day discharge” defined as same-day discharges (without overnight stay), absence of complication above Clavien-Dindo grade II, and no readmission for any reason within 30 days postoperatively.

Results showed that all patients treated as same-day discharge surgical patients were successfully and safely discharged without any complications. 93% of patients admitted on the same-day adrenalectomy (SDA) pathway were discharged within 23 hours of admission. No patients in any group suffered complications.

With high patient satisfaction, no complications, and high efficacy, this paper provides a promising foundation for further advancement in care for patients undergoing laparoscopic adrenalectomy. Given that fewer than 200 patients have been included in studies related to same-day adrenalectomies, this study contributes a small but impactful addition to this topic. It is the among the first studies to prospectively follow patients after same-day adrenalectomy and is the first to provide matched patients with planned admission as a comparison group.

Overall, the authors defined the methods of this investigation clearly, including the same-day adrenalectomy pathway itself. This allows for easy replication in the future, increasing the feasibility of expansion to a multi-center study and validation of the results of this smaller study. Though the results from this study are optimistic, they are limited by lack of generalizability. The study was performed at a center with three experienced high-volume surgeons. In a recent review, high-volume adrenal surgeons were defined as performing six or more adrenalectomies per year, and had much more favorable outcomes than those performing fewer than six [5]. However, within the United states, about 80 % of adrenalectomies are performed by low-volume surgeons [5]. Additionally, the same-day adrenalectomy pathway was carried out by expert anesthesiologists and skilled nurses with capacity to call patients at home. These resources are not likely available at every hospital or surgical center. Thus, the same-day adrenalectomy pathway may not be appropriate or feasible in smaller centers. However, should a center match these criteria, patients may be screened for the same-day adrenalectomy pathway as appropriate.

Patient selection is crucial to the success of this trial. Patients were only offered same-day adrenalectomy if they fulfilled specific criteria including BMI <33, low ASA, age below 70 years, well-controlled hypertension on a single agent, correction of hyperkalemia, and support at home. Additionally, only patients motivated to participate in same-day discharge were selected. While these criteria are reasonable for any planned same-day procedure, care must be taken to ensure that future patients fit within these confines. More medically complex patients were not included in the study, and so consideration for these patients should be taken to determine suitability for a fast-tracked discharge, whether admitted or not. Further, case time was significantly shorter in those enrolled in the same-day adrenalectomy cohort, which may imply that patients selected for same-day adrenalectomy were less complex than those assigned inpatient admission, despite matching. Patient selection may limit the application of the same-day adrenalectomy pathway, though with appropriate screening, the same-day adrenalectomy pathway was shown to be safe in a specific population.

Overall, this study is a meaningful steppingstone for further investigation, providing methodology and evidence of safety, justifying its progression to larger studies. Its success in prospectively proving safety of same-calendar-day discharge as opposed to the conventional three-day hospital admission average is significant. Patients in the inpatient arm were also able to be discharged sooner than historical examples in matched patients. However, its single-center design with small sample size limits its impact. Rigorous selection criteria may further narrow its application. Before this pathway can be widely adopted, further investigation is needed, ideally with larger and more diverse sample populations.

In conclusion, the results of this study justify further studies of same-day adrenalectomy with evidence of safety and patient satisfaction. Based on this small pilot investigation, as long as the selection process is thorough, there is potential for same discharge in patients undergoing laparoscopic adrenalectomy. Further studies should expand upon this by including resource-limited or lower volume settings, more diverse patients, and a larger sample size.

## Funding sources

Dr. James has funding through the Martin and Diane Trust Career Development Chair position and none of these funds were used in the process of drafting this commentary.

## Credit authorship contribution statement

Dr. James and Dr. Bogdanovski participated in commentary development and editing.

## Declaration of competing interest

None.

## References

[1] Brunt L.M. (2002). The positive impact of laparoscopic adrenalectomy on complications of adrenal surgery. Surg Endosc.

[2] Castillo O., Sánchez-Salas R., Vidal I. (2008). Laparoscopic adrenalectomy. Minerva Urol Nefrol.

[3] Gonzalez R., Smith C.D., McClusky D.A. (2004). Laparoscopic approach reduces likelihood of perioperative complications in patients undergoing adrenalectomy. Am Surg.

[4] Limberg J., Ullmann T.M., Gray K.D. (2019). Laparoscopic adrenalectomy has the same operative risk as routine laparoscopic cholecystectomy. J Surg Res.

[5] Kazaure H.S., Sosa J.A. (2019). Volume-outcome relationship in adrenal surgery: a review of existing literature. Best Pract Res Clin Endocrinol Metab.

